# The Impact of Immediate Verbal Feedback on the Improvement of Swimming Technique

**DOI:** 10.2478/hukin-2014-0042

**Published:** 2014-07-08

**Authors:** Krystyna Zatoń, Stefan Szczepan

**Affiliations:** 1 University School of Physical Education in Wroclaw, Poland, Department of Swimming.

**Keywords:** motor control, verbal information, immediate verbal feedback, swimming technique

## Abstract

The present research attempts to ascertain the impact of immediate verbal feedback (IVF) on modifications of stroke length (SL). In all swimming styles, stroke length is considered an essential kinematic parameter of the swimming cycle. It is important for swimming mechanics and energetics. If SL shortens while the stroke rate (SR) remains unchanged or decreases, the temporal-spatial structure of swimming is considered erroneous. It results in a lower swimming velocity. Our research included 64 subjects, who were divided into two groups: the experimental – E (n=32) and the control – C (n=32) groups. A pretest and a post-test were conducted. The subjects swam the front crawl over the test distance of 25m at Vmax. Only the E group subjects were provided with IVF aiming to increase their SL. All tests were filmed by two cameras (50 samples•s-1). The kinematic parameters of the swimming cycle were analyzed using the SIMI Reality Motion Systems 2D software (SIMI Reality Motion Systems 2D GmbH, Germany). The movement analysis allowed to determine the average horizontal swimming velocity over 15 meters. The repeated measures analysis of variance ANOVA with a post-hoc Tukey range test demonstrated statistically significant (p<0.05) differences between the two groups in terms of SL and swimming velocity. IVF brought about a 6.93% (Simi method) and a 5.09% (Hay method) increase in SL, as well as a 2.92% increase in swimming velocity.

## Introduction

Swimming is a highly complex motor skill and its acquisition or efforts towards any technical improvement require a specific procedure of bilateral information transmission between the swimmer and the coach. Indeed, specific environmental factors make it more difficult to exchange information (head immersed in water, swimming cap, ambient noise) and this leads to errors. From the standpoint of the information theory, swimming acquisition and technique improvement are thus considered challenging (Adams, 1971; Schmidt, 1975; Figueiredo et al., 2011; Stanula et al., 2012).

The present work attempted to minimize the interference in didactic verbal communication during the instructional process of improving swimming technique. It was assumed that a verbal message prepared in accordance with the criteria of the information theory, provided to the learner in real time, immediately in reaction to a specific element of a movement structure, is a factor minimizing the interference in the information flow. Such a form of information transmission helps eliminate in advance the stimuli which are not essential to the execution of an exercise (Marteniuk, 1976; Lee et al., 1994). In physical education, this preventive method entails the anticipation of errors, which may occur in a specific exercise due to its early acquisition or further attempts to improve, while at the same time eliminating their possible causes (Lee et al., 1993). This is why real time (immediate) feedback is more efficient in dealing with such errors (Marteniuk, 1976; Magill, 1993; Schmidt and Lee, 1999).

The above assumptions are based on the physiological structure of human memory, where different types are classified depending on how long information is stored (Atkinson and Shiffrin, 1968). In short-term memory, data is processed but it can only contain a limited amount of information (Miller, 1956). The quantity of information conveyed to the learner must be limited. It is worth noting that unimportant data is not stored (sometimes permanently) within long-term memory, which gathers in experiences and motor habits; hence, the necessity to provide the learner with real time information and accurate instructions.

Following the findings of international research, it has become necessary to devise a means of transmitting real-time verbal information to the learner.

Some authors believe that verbal information is the most efficient form of didactic communication (Landin, 1996; More and Franks, 1996; Zatoń, 2010). However, due to such disruptive factors as noise in a swimming pool, it cannot be used to its full potential in the aquatic environment. Therefore, it has become possible, but with the aid of modern technology which enables the teacher to communicate with the learner in order to prevent and immediately eliminate potential errors in the movement structure.

The focus of the research was error prevention in swimming. Reduced stroke length was identified as an erroneous element of its temporal-spatial structure (relative to the stroke rate). Stroke length is the horizontal displacement of the swimmer’s body in one stroke cycle (Hay et al., 1983) while the stroke rate is the number of full cycle motions executed in one time unit (Hay et al., 1983).

Error prevention consisted of providing real time feedback to the swimmers in order to increase stroke length. International research has demonstrated that stroke length modulation is central to swimming technique improvement. It has been assumed that when stroke length decreases while stroke rate remains unchanged or decreases, the temporal-spatial structure of swimming is erroneous and results in lower swimming velocity (Ballreich, 1976).

The importance of stroke length modulation has also been demonstrated by authors who claim that low stroke length is the most frequent cause of low swimming velocity (Craig et al., 1985; Hay et al., 1983). It must be added that the ability to maintain constant stroke length at its maximum value relative to the locomotive movement frequency helps minimize fluctuations of inner cyclic velocity (Schnitzler et al., 2010; Fernandes et al., 2012). An increase in energy costs was triggered by significant fluctuations in inner cyclic velocity (Kolmogorov and Duplischeva, 1992; Alves et al., 1996; Barbosa et al., 2005).

Thus, an increase in stroke length (relative to the stroke rate) significantly improves the biomechanical characteristics of a swimming technique and its physiological variables relative to energy cost. Therefore, there is a need for the implementation of pre-emptive measures aimed at increasing the stroke length (and consequently decreasing the number of movement cycles), which along with greater locomotive velocity, improves swimming economy. That alone often constitutes the primary objective of teaching or technique improvement training.

Given that an efficient action results, to a lesser or greater extent, in the accomplishment of an objective (Doran, 1981), in order to verify the efficiency of immediate verbal feedback in the improvement of technique, the authors of this work analyzed the changes in stroke length and the stroke rate (the kinematic parameters of a movement cycle) occurring as a result of verbal feedback.

The authors attempted to define the impact of immediate verbal feedback on stroke length modifications. Our objective consisted of verifying the research hypothesis. It was assumed that immediate verbal feedback significantly influenced stroke length (H1).

## Material and Methods

The experiment was conducted in two groups (control and experimental groups) in order to examine the changes resulting from the introduction of the experimental factor (independent variable) in the experimental group. A pretest and a post-test were carried out. Data analysis was performed at the ISO 9001:2009 certified Aquatic Exercise Research Laboratory. All subjects gave written informed consent for participation in this research. The study was also approved by the University’s School of Physical Education in Wroclaw ethics committee.

### Research groups

64 male subjects agreed to participate in the experiment and met the conditions necessary to define the inter-group differences. No significant interactions between the sports level and gender, performance, anthropometrics, kinematics and energetic parameters in young swimmers were reported (Morais et al., 2013). Therefore, the subjects were assigned into two homogeneous groups. The subjects were selected based on the following criteria of resemblance: (1) Gender (men); (2) Group (students); (3) Age (control group 20.44, ±0.67 years; experimental group 20.53, ±0.92 years); (4) Front crawl technical skill, evaluated by two experts (two independent referees) using precise point system criteria; (5) Time over 25 meters for front crawl before the initial test. The student’s t-test did not show any statistically significant differences between the groups, which was confirmed by the homogeneity of their swimming times (t =−0,142; p=0,887; p<0,05); (6) Somatic parameters: body mass (control group 74.97, ±9.58 kg; experimental group 74.31, ±6.72 kg), body height (control group 1.79, ±0.06 m; experimental group 1.80, ±0.06 m) - these constitute objective criteria pointing to a similar capacity of the subjects to generate propulsion, which influences the kinematic variables of the swimming cycle. In order to compare the groups’ somatic variables, it was decided that the standard deviation could not exceed 10% of the average body height value (control group 10% 0.18 m; experimental group − 10% 0.18 m). Implementing the above requirements was necessary to make sure the motor potentials of the subjects were similar. The two homogenous research groups were constituted – as the control group (n=32 subjects) and the experimental group (n=32 subjects).

### Verbal message

Verbal messages were in our research project the sole source of information available to the swimmers. Before the start of the experiment, pilot tests focused on elaborating the verbal message to be used (Zatoń and Szczepan, 2012). It had to be consistent with the objective of increasing stroke length and it was expected to make the subjects aware of the objective. Since during the acquisition process, the learners’ hearing perception is limited (Lee et al., 1994) the structure of verbal communication was put to scrutiny. To rationalize verbal information the criteria of efficient didactic communication was applied (Zatoń, 2010). The terms used in the verbal message were confirmed in advance to have carried the same meaning for the subjects and the researchers. Given the conditions in which our experiment was carried out, the researcher uttered successive messages until the swimmer’s stroke length actually increased. In addition, when the research groups were being constituted, we made sure that the subjects were physically apt to execute every test procedure. We also took account of the structure of the human memory which can only store a limited quantity of information (Miller, 1956). Therefore it was necessary to eliminate any unnecessary data which might have been transmitted to the swimmer, in order to preserve the short-term memory potential. That is how the verbal message – “reach out further” – was formulated. It is labeled verbal feedback in the present work (independent variable). Since it was provided to the subjects as they were in the process of swimming, it was categorized as immediate or real-time (Schmidt and Lee, 1999).

### Procedure

The experiment consisted of 4 front crawl swimming trials. Before starting out, the subjects assumed a motionless horizontal position. The first and the second trial for both the control and experimental groups consisted of swimming 25 meters at maximum speed without the provision of any real-time verbal information. The first and the second trial were considered a pretest. The third and the fourth trial in the experimental group also consisted of swimming over 25 meters at maximum speed, but with immediate verbal feedback, which aimed to increase the swimmers’ stroke length (independent variable). The third and the fourth trial were considered a posttest. The verbal messages were provided to the swimmers by the same experimenter. In the control group, no information whatsoever was transmitted to the swimmers. In order to minimize the fatigue factor, the subjects swam all the trials with a rest heart rate established in the 5th minute following a 15-minute warm up.

The maximum speed condition implemented in our work was based on the assumption that stroke length is an individual variable dependent on swimming velocity (Craig and Pendergast, 1979). In order to ensure the subjects’ full cooperation (swimming at maximum speed), they were advised that the average swimming time over 25 meters in trials 1 and 2 (pretest) should not exceed the average swimming time in trials 3 and 4 (post-test) by more than 10%. The average swimming time value in trials 3 and 4 relative to the average swimming time value in trials 1 and 2 is described by the following equation (1):

(1) $$t_{3,4/1,2}=100-\frac{t_{3,4}}{t_{1,2}}\times 100$$

Because all the swimmers were motivated to reach their maximum swimming speed (with a tolerance of 10%) the results obtained can be considered reliable.

In order to record data pertaining to the kinematic variables of the swimming cycle (stroke length, stroke rate) the subjects were filmed under and above the water surface. All tests were recorded by two 50 frames per s-1 cameras. One waterproof camera (1C) was fixed under water at the center of the pool. The camera lens axis was perpendicular to the swimmer’s body, which allowed for the largest possible picture format to be obtained. The underwater (1C) camera was used to record each subject’s movement cycle over a 15-meter so called clean swimming area (excluding the 5-meter start and turn areas). The other camera (2C) was fixed over the center of the pool, and also enabled to record the movement cycles of each swimmer over the 15-meter clean swimming area.

**Figure 1:**
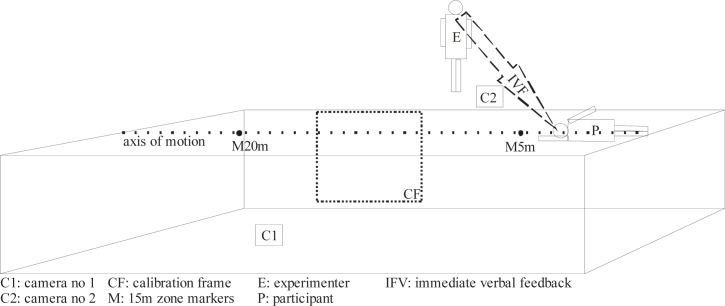
Measurement chain

The actual spatial dimensions were defined using two coordinate systems. The first system was the 2m × 2m scale calibration frame. The calibration frame was placed vertically in the swimmer’s movement plane, in a way that did not disrupt the trials. Since the camera lens axis was perpendicular to the swimmers, the camera eye captured the largest calibration system possible. Markers with contrasting colors to the ambient environment placed on both upper limbs in the radiocarpal joint axis and on the center of the head (Plagenhoef, 1971) enabled us to follow the displacement of the subject’s body. The center of the radiocarpal joint corresponds to the midpoint of the distance between the styloid process of the radius and the lentiform bone, while the center of the head in the saggital plane is a point on the temple near the auditory meatus (Zatsiorsky, 1998). Another coordinate system, comprised of two markers with contrasting colors to the ambient environment, was placed on the 5th and the 20th meter lines of the pool to delineate the 15-meter clean swimming area. Each trial was executed in homogenous conditions – temperature of 25–28°C, illumination of 600 lux, in accordance with FINA (Fédération Internationale de Natation) guidelines. Figure 1 illustrates the experiment.

### Experimental device

A wireless communication set (Wrocław University of Technology, PL) was used courtesy of the ISO 9001:2009 certified Aquatic Exercise Research Laboratory. It enabled the researcher to transmit verbal feedback to the swimmer, the latter having tucked a waterproof receiver behind the goggle strap and put on waterproof earphones (Picture 1a). The researcher was also equipped with a transmitter and a microphone (Picture 1b). The sound was transmitted wirelessly to the participant. Picture 1 a, b shows the receiver and the transmitter.

### Kinematic variables of the swimming cycle

#### Stroke length

Stroke length is defined as the horizontal displacement of the swimmer’s body in one movement cycle. To improve the accuracy of the results, two different methods were employed (the Simi and the Hay methods) to analyze the video material and establish the accuracy of the stroke length values.

Firstly (the Simi method), we used the SIMI Motion 2D (SIMI Reality Motion Systems 2D GmbH, Germany) movement analysis software. All manufacturer recommendations were met during the movement recording sessions.

The stroke length parameter was defined (Picture 2) based on the horizontal displacement of the marker fixed on the swimmer’s head during the interval between the preliminary sensorimotor stage (a) (getting a grip on the water) and the moment when the hand, having executed the propulsive movement and the preparation phase, goes back to the initial position (a) (Chollet et al., 1997). Only strokes performed within the 15-meter clean swimming area were analyzed.

Secondly, the Hay method was applied (Hay et al., 1983) to calculate average stroke length and the number f movement cycles executed by the participants within the 15-meter clean swimming area.

(2) $$\mathrm{SL}=m\cdot {\mathrm{cycle}}^{-1}$$

SL - stroke length, m - distance covered, cycle - number of movement cycles

#### Stroke rate

A stroke rate is defined as the number of full movement cycles performed in a time unit (Hay et al., 1983), as described by the formula (3). The number of movement cycles was established using the SIMI Motion 2D movement analysis software. Only those movement cycles performed within the 15-meter clean swimming area were taken into account.

(3) $$\mathrm{SR}=\mathrm{cycle}\cdot s^{-1}$$

SR - stroke rate, cycle - number of movement cycles, s - time to cover the distance

### Relationships between stroke length and the stroke rate

Stroke length is assessed relative to the frequency of propulsive cycles. That is why the present work also tackles the relationships between stroke length and the stroke rate as described by Ballreich (1976).

### Swimming velocity

The average horizontal swimming velocity over 15 meters was defined based on the direct movement analysis performed using the SIMI motion 2D software (SIMI Reality Motion Systems 2D GmbH, Germany).

### Data collection

Data was collected using the following formulae (4 – 11):

The average stroke length in trials 1 and 2 was determined by the following equation:

(4) $$l_{1,2}=\frac{l_{1}+l_{2}}{2}$$

Likewise, average stroke length in trials 3 and 4 was determined by the following equation:

(5) $$l_{3,4}=\frac{l_{3}+l_{4}}{2}$$

The percentage weight of average stroke length in trials 3 and 4 relative to trials 1 and 2 was determined by the following equation:

(6) $$l_{3,4/1,2}=100-\frac{l_{3,4}}{l_{1,2}}\times 100$$

The differences in stroke length between trials 1, 2 and 3, 4 were determined by the following equation:

(7) $$\Delta l=l_{1,2}-l_{3,4}$$

The average stroke rate in trials 1 and 2 was determined by the following equation:

(8) $$f_{1,2}=\frac{f_{1}+f_{2}}{2}$$

Likewise, for trials 3 and 4, the average stroke rate was determined by the following equation:

(9) $$f_{3,4}=\frac{f_{3}+f_{4}}{2}$$

The percentage weight of the average stroke rate in trials 2 and 4 relative to trials 1 and 2 was determined by the following equation:

(10) $$f_{3,4/1,2}=100-\frac{f_{3,4}}{f_{1,2}}\times 100$$

The differences in the stroke rate between trials 1, 2 and 3, 4 were determined by the following equation:

(11) $$\Delta f=f_{1,2}-f_{3,4}$$

### Statistical analysis

Statistical analysis was conducted under Statistica 9.0 software (StatSoft, USA) and the level of significance was set at p<0.05. The presented results are the averaged values and the differences between those established during research trials. The Student’s t-test for independent samples was performed to ascertain possible statistically significant pretest and post-test differences within the research groups. Indeed, the student’s t-test evidenced statistically significant differences between the groups in terms of stroke length (the Hay and the Simi methods) and horizontal swimming velocity, which proved the pertinence of the said parameters when it came to comparing the research groups (Table 1). Table 2 illustrates the differences between the groups (in percentage terms and parameter units). A repeated measures analysis of variance ANOVA was performed to determine whether pretest and post-test differences were significant between the research groups. The Tukey post-hoc test was conducted to assess precise differences between the groups (Table 3).

## Results

Table 1 presents inter-group differences -parameters under consideration in research trials assessed by the student’s t-test. Table 2 constitutes a comparison of the study groups. The parameters distinguishing the study groups are given is percentage and real values.

Precise pretest and post-test differences between the groups were established using the repeated measures analysis of variance ANOVA with the post-hoc Tukey range test. In the post-tests, the experimental group’s differ to a statistically significant degree from those in the control group in stroke length (the Hay and the Simi methods) and swimming velocity. With regard to the stroke rate, the difference is not statistically significant between the two groups (Table 3).

## Discussion

The aim of this study was to determine whether verbal information transmitted instantaneously affects the selected element of time-space structure of swimming motility, which is a swimming stroke. It was assumed that the instantaneous transfer of verbal information will modify the movement in the water - changing length of the swimming stroke. Adjusting length of the swimming step prevents the formation of an error in the time-space swimming movement. The hypothesis was that the verbal information transmitted instantaneously would allow for an extension of the swimming step. It was proved that the teacher or coach using properly selected verbal information, prepared in accordance with the criteria of effective educational communication and transferred immediately to the student or competitor, is able to regulate the swimming movement in real time, preventing the errors in the structure of movement.

Error prevention and elimination is an essential role of the teacher (Newell et al., 1985; Zatoń, 2010). Feedback allows for error correction in the short-term memory or prevention of their occurrence (Lee et al., 1994; Schmidt and Lee, 2005). Therefore, in the present study, verbal information was transmitted instantaneously. The verbal transfer was directed at increasing the swimming stroke with the intention of preventing the occurrence of an error in the space-time structure of the swimming movement. Shortening of the swimming stroke length in relation to the constant or decreasing frequency of cyclical movements is an error in the space-time structure of the swimming movement. Shortening of the swimming stroke length results in a decreased swimming speed (Ungerechts and Theismann, 1995; Hay, 2002). It was therefore concluded that the verbal control of the swimming stroke, when aiming at its extension, will cause an increase in swimming speed. The introduction of instantaneous verbal information resulted in the lengthening of the swimming stroke and an increase in swimming speed in the experimental group. In addition, another finding of this study is that the use of verbal feedback prevented the occurrence of errors in the space-time structure of the swimming movement.

Understanding of verbal information requires it to be properly prepared in accordance with the agreed criteria of educational communication. The created protocol of verbal information was designed to clarify the objective of completing a task (the elongation of the swimming stroke). It is recognized that the criteria which need to be met for effective communication contribute to optimizing the teacher’s educational information. These include the criterion of syntax, semantics and pragmatics. Other criteria for effective communication are as follows: quantity, frequency and accuracy of the transmitted information (Williams and Hodges, 2005).

Research conducted by Siedentop (1997), Siedentop and Eldar (1990) showed that teachers, during teaching of movement activities, provide information with high frequency. The authors also showed that teachers provide information during physical education classes primarily through verbalization, however, more frequently while learning new tasks, rather than when improving already acquired skills. Yerg (1990) showed that during teaching, an increase in the number of positive information affects the degree to which the task is mastered and the students’ level of interest in the lesson. According to the authors, a higher frequency of transmitted information impacts teaching results.

An additional criterion that determines the effectiveness of the teaching process and motor skills improvement is the content of the transmitted information (Magill, 1994; Wulf and Shea, 2002). Content of the conveyed feedback in this experiment was deliberately limited due to the conditions of the experiment as well as by the nature of instantaneous messages.

Sadowski et al. (2011) evaluated the effectiveness of the learning of complex motor activities with the use of various types of verbal information. The authors argued that verbal feedback was more effective when it included a combination of praise and constructive advice for error correction. This claim was also confirmed by Tzetzis and Votsis (2006). The results of their research suggest that feedback must include information on the mistakes and how to eliminate them. In addition, Zatoń (2010) states that verbal feedback is highly effective in eliminating errors when the content consists of simple sentences or single words.

The aquatic environment generates many additional stimuli in the process of teaching and learning to swim. Many authors have indicated that the type of information provided by the teacher (verbal, non-verbal) influences the effectiveness of both teaching and learning to swim (Zatoń, 2010). Numerous pieces of information deriving from the organ of sight, hearing, and depth perception can result in the learner’s adverse reaction. The aquatic environment can generate difficulties in educational perception such as the distance between the teacher and learner or dominant noise. Difficulties in the perception of teaching can contribute to error occurrence in motor activity. Therefore, in this study, communication tools were used to eliminate disturbances. The immediate elimination of errors prevents their reinforcement (Schmidt and Lee 2005). Due to the instantaneous error elimination, it is possible to consolidate the correct structure of movements in the long-term motor memory and form motor habit.

Used in the past, communication tools aimed primarily at informing the swimmer about swimming speed (Gonzalez et al., 2002; Seifert and Chollet, 2005; Vezos et al., 2007; Perez et al., 2009) as well as on the strength achieved on the swimming ergometer (Petriaev and Kleshnev, 2006).

Instantaneous verbal communication, despite the development of devices that enable non-verbal communication, is considered to be the most effective form of educational communication (Zatoń, 2010).

In view of the above, the instantaneous verbal information optimizes swimming technique. The influence of the quantity and frequency of the conveyed information is one of the main scientific interests in the process of optimizing swimming technique and the improvement of motor function (Magill, 1992; Silverman, 1994; More, 1996; Sanchez and Bampouras, 2006).

## Conclusions

The problem undertaken in this study refers to such aspects as: a) removing barriers in communication when teaching, b) providing instantaneous information regarding current motor action to the swimmer c) prevention of errors in the space-time structure of swimming motility. In the opinion of the authors of this paper, a proposed method of instantaneous communication will enrich the technology of information transmission. This method can be used by educators in physical education as well as for recreation and swimming coaches in competitive sport. In addition, the use of instantaneous verbal communication method allows for the immediate breakdown of communication barriers, prevents error occurrence and creates conditions for the development of normal motor habits. A direct implementation of the results of this study to the practice of teaching would increase the effectiveness of teaching methods, as well as economise movement techniques. Due to the extent of the issues related to educational communication during the teaching process and the improvement of swimming movements, research in this area should be continued.

## Figures and Tables

**Picture 1:**
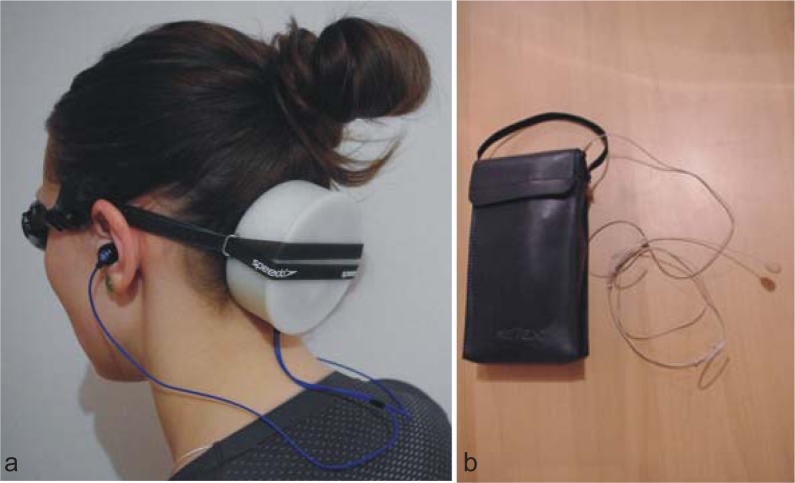
Waterproof communication set used to transmit verbal feedback, a) participant with the receiver, b) experimenter’s transmitter

**Picture 2:**
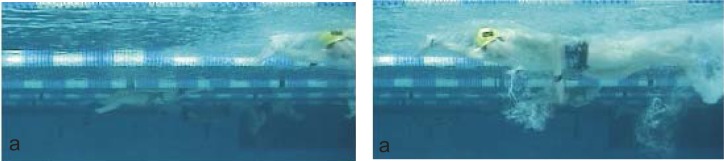
Stroke length measurement procedure (between the preliminary a stages) (Chollet et al., 1997)

**Table 1:** Inter-group differences - parameters under consideration in research trials assessed by the Student’s t-test

Parameter	Measurement 1,2 (pretest) 3,4 (posttest)	t	df	Significance (two sided)
Stroke length (Simi method)	1.2	0,689	62	0,494
	**3.4**	**−2.143**	**62**	**0,036**
Stroke length (Hay method)	1.2	−0.915	62	0,364
	**3.4**	**−2.403**	**62**	**0,019**
Stroke rate	1.2	0.841	62	0,404
	3.4	0.402	62	0,689
Horizontal swimming velocity	1.2	−1.743	62	0,086
	**3.4**	**−2.966**	**62**	**0,004**

Statistically significant differences alpha=0.05 (p<0.05) marked in bold

**Tabela 2:** Comparative characteristics of the two research groups (in percentage terms and units of parameters)

Parameter	Group	N	*x̄*		±		Min		Max	
Stroke length (Simi method) (m)	C	32	−1.89%	−0.03	7.34%	0.11	−17.17%	−0.26	14.48%	0.17
	E	32	6.93%	0.12	6.11%	0.11	−2.61%	−0.03	23.44%	0.46
	Overall	64	2.52%	0.04	8.04%	0.13	−17.17%	−0.26	23.44%	0.46
Stroke length (Hay method) (m)	C	32	−0.13%	0.00	5.34%	0.09	−16.12%	−0.30	12.76%	0.22
	E	32	5.09%	0.09	6.88%	0.13	−9.10%	−0.14	20.75%	0.47
	Overall	64	2.48%	0.05	6.65%	0.12	−16.12%	−0.30	20.75%	0.47
Stroke rate (cycle·s^−1^)	C	32	0.97%	0.01	7.37%	0.11	−22.85%	−0.39	12.63%	0.19
	E	32	2.21%	0.03	6.75%	0.09	−10.62%	−0.13	15.79%	0.26
	Overall	64	1.59%	0.02	7.04%	0.10	−22.85%	−0.39	15.79%	0.26
Horizontal Swimming velocity (m·s^−1^)	C	32	−1.05%	−0.02	5.66%	0.07	−12.36%	−0.16	10.02%	0.13
	E	32	2.92%	0.04	3.42%	0.04	−2.54%	−0.04	11.78%	0.14
	Overall	64	0.93%	0.01	5.06%	0.06	−12.36%	−0.16	11.78%	0.14

A negative result denotes an average value decrease of - n.

A positive result denotes an average value increase of - n.

**Table 3:** Repeated measures analysis of variance ANOVA

Parameter	F	p
stroke length (Simi method).	29,157	**0,001**
stroke length (Hay method).	11,624	**0,001**
stroke rate	0,697	0,407
average horizontal swimming velocity over 15 meters.	12,969	**0,001**

Statistically significant differences alpha=0.05 (p<0.05) marked in bold
